# Complete Genome Sequence and Methylome Analysis of *Acinetobacter calcoaceticus* 65

**DOI:** 10.1128/genomeA.00060-17

**Published:** 2017-03-23

**Authors:** Alexey Fomenkov, Tamas Vincze, Sergey K. Degtyarev, Richard J. Roberts

**Affiliations:** a New England BioLabs, Ipswich, Massachusetts, USA; b SibEnzyme Ltd., Novosibirsk, Russia

## Abstract

*Acinetobacter calcoaceticus* 65 is the original source strain for the restriction enzyme Acc65I. Its complete sequence and full methylome were determined using single-molecule real-time (SMRT) sequencing.

## GENOME ANNOUNCEMENT

The bacterial strain* Acinetobacter calcoaceticus* 65 (recently renamed *Acinetobacter junii* 65) was originally isolated from a limnetic water sample and is now housed in the New England BioLabs culture collection (NEB1023). It is the original source of the Type II restriction enzyme Acc65I, identified as a neoschizomer of KpnI. The Acc65 restriction enzyme recognizes and cleaves the DNA sequence G**↓**GTACC, producing 5′ cohesive ends, in contrast to KpnI, which cleaves the same DNA sequence but leaves a 3′ extension GGTAC**↓**C (1, 2).

Genomic DNA from a culture of* A. calcoaceticus* 65 was purified using a modified Qiagen method and the genome sequenced using the Pacific Biosciences (PacBio) RSII sequencing platform. Briefly, SMRTbell libraries were constructed from a genomic DNA sample sheared to an average size of ~10 to 20 kb using the G-tubes protocol (Covaris, Woburn, MA, USA), end repaired, and ligated to hairpin adapters. Incompletely formed SMRTbell templates were digested with a combination of exonuclease III and exonuclease VII (New England BioLabs; Ipswich, MA, USA). Genomic DNA fragments and SMRTbell library qualification and quantification were performed using a Qubit fluorimeter (Invitrogen, Eugene, OR) and a 2100 Bioanalyzer (Agilent Technologies, Santa Clara, CA). One 20-kb SMRTbell library was prepared according to the modified PacBio sample preparation protocols, including additional separation on a BluePippin, and sequenced using C4-P6 chemistry and three single-molecule real-time (SMRT) cells with a 240-min collection time. Sequencing reads were processed, mapped, and assembled by the Pacific Biosciences SMRT Analysis pipeline, using the HGAP3 protocol, and polished using Quiver (3) to yield 632.2 Mb of sequence data, which were assembled into a single closed circular genome of 3,377,773 bp with 134-fold coverage. The assembled sequence was annotated using the NCBI Prokaryotic Genome Annotation Pipeline (PGAP) and has been deposited at DDBJ/EMBL/GenBank (Table 1).

**TABLE 1:** Summary of methyltransferases identified in *Acinetobacter calcoaceticus* 65

Motif^a^	Assigned or predicted	Methylation type	Restriction modification type
GG**TA**CC	M.Acc65I	m6A	II beta
CT**A**GNNNNNNN**T**GAA	M.Acc65II	m6A	I
CNNT**A**YNNNNNN**T**CTT	M.Acc65III	m6A	I gamma
G**A**GNNNNN**T**CG	M.Acc65IV	m6A	I gamma
GACGC**A**	M.Acc65V	m6A	II G, S gamma
C**T**KM**A**G	M.Acc65VI	m6A	II gamma

^a^ Modified bases are highlighted in bold.

The advantage of the PacBio sequencing platform is its ability to detect the epigenetic state of sequenced DNA, which allows for the identification of modified nucleotides and their corresponding motifs. Epigenetic modification at each nucleotide position was measured as kinetic variations (KVs) in the nucleotide incorporation rates, and methylated motifs were deduced from the KV data (4–6). Six DNA methyltransferase recognition motifs were detected, each containing m6A modifications. The motifs were then matched with methyltransferase genes in the genome, and the results are shown in the table below. They have also been deposited in REBASE (7).

### Accession number(s).

The complete genome sequence of the *Acinetobacter calcoaceticus* 65 is available in GenBank under the accession number CP019041 (*Acinetobacter junii* 65).
